# Multi-omics analysis of overexpressed tumor-associated proteins: gene expression, immunopeptide presentation, and antibody response in oropharyngeal squamous cell carcinoma, with a focus on cancer-testis antigens

**DOI:** 10.3389/fimmu.2024.1408173

**Published:** 2024-07-29

**Authors:** Tsima Abou Kors, Matthias Meier, Lena Mühlenbruch, Annika C. Betzler, Franziska Oliveri, Martin Bens, Jaya Thomas, Johann M. Kraus, Johannes Doescher, Adrian von Witzleben, Linda Hofmann, Jasmin Ezic, Diana Huber, Julian Benckendorff, Thomas F. E. Barth, Jens Greve, Patrick J. Schuler, Cornelia Brunner, Jonathan M. Blackburn, Thomas K. Hoffmann, Christian Ottensmeier, Hans A. Kestler, Hans-Georg Rammensee, Juliane S. Walz, Simon Laban

**Affiliations:** ^1^ Department of Otorhinolaryngology and Head and Neck Surgery, Ulm University Medical Center, Ulm, Germany; ^2^ Department of Immunology, Institute for Cell Biology, Eberhard Karls University of Tübingen, Tübingen, Germany; ^3^ Department of Peptide-based Immunotherapy, Eberhard Karls University and University Hospital Tübingen, Tübingen, Germany; ^4^ Cluster of Excellence iFIT (EXC2180) “Image-Guided and Functionally Instructed Tumor Therapies”, University of Tübingen, Tübingen, Germany; ^5^ German Cancer Consortium (DKTK), Partner Site Tübingen, Tübingen, Germany; ^6^ Core Facility Immune Monitoring, Ulm University Medical Center, Ulm, Germany; ^7^ Core Facility Next Generation Sequencing, Leibniz Institute on Aging - Fritz Lipmann Institute, Jena, Germany; ^8^ Cancer Sciences Unit, University of Southampton, Faculty of Medicine, Southampton, United Kingdom; ^9^ Institute of Medical Systems Biology, Faculty of Medicine, Ulm University, Ulm, Germany; ^10^ Department of Otolaryngology, Augsburg University Hospital, Augsburg, Germany; ^11^ Institute of Pathology, Ulm University Medical Center, Ulm, Germany; ^12^ Surgical Oncology Ulm, i2SOUL Consortium, Ulm, Germany; ^13^ Institute of Infectious Disease and Molecular Medicine, Faculty of Health Sciences, University of Cape Town, Cape Town, South Africa; ^14^ Institute of Systems, Molecular and Integrative Biology, Liverpool Head and Neck Center, University of Liverpool, Faculty of Medicine, Liverpool, United Kingdom; ^15^ Clinical Collaboration Unit Translational Immunology, Department of Internal Medicine, University Hospital Tübingen, Tübingen, Germany

**Keywords:** oropharyngeal squamous cell carcinoma (OPSCC), HLA, cancer testis antigens (CTA), tumor-associated peptide (TAP), antibody response (AR)

## Abstract

**Introduction:**

The human leukocyte antigen complex (HLA) is essential for inducing specific immune responses to cancer by presenting tumor-associated peptides (TAP) to T cells. Overexpressed tumor associated antigens, mainly cancer-testis antigens (CTA), are outlined as essential targets for immunotherapy in oropharyngeal squamous cell carcinoma (OPSCC). This study assessed the degree to which presentation, gene expression, and antibody response (AR) of TAP, mainly CTA, are correlated in OPSCC patients to evaluate their potential as immunotherapy targets.

**Materials and methods:**

Snap-frozen tumor (N_Ligand/RNA_=40), healthy mucosa (N_RNA_=6), and healthy tonsils (N_Ligand_=5) samples were obtained. RNA-Seq was performed using Illumina HiSeq 2500/NovaSeq 6000 and whole exome sequencing (WES) utilizing NextSeq500. HLA ligands were isolated from tumor tissue using immunoaffinity purification, UHPLC, and analyzed by tandem MS. Antibodies were measured in serum (N_Ab_=27) utilizing the KREX™ CT262 protein array. Data analysis focused on 312 proteins (KREX™ CT262 panel + overexpressed self-proteins).

**Results:**

183 and 94 of HLA class I and II TAP were identified by comparative profiling with healthy tonsils. Genes from 26 TAP were overexpressed in tumors compared to healthy mucosa (LFC>1; FDR<0.05). Low concordance (r=0.25; p<0.0001) was found between upregulated mRNA and class I TAP. The specific mode of correlation of TAP was found to be dependent on clinical parameters. A lack of correlation was observed both between mRNA and class II TAP, as well as between class II tumor-unique TAP (TAP-U) presentation and antibody response (AR) levels.

**Discussion:**

This study demonstrates that focusing exclusively on gene transcript levels fails to capture the full extent of TAP presentation in OPSCC. Furthermore, our findings reveal that although CTA are presented at relatively low levels, a few CTA TAP-U show potential as targets for immunotherapy.

## 1 Introduction

Oropharyngeal squamous cell carcinoma (OPSCC) is a type of head and neck squamous cell carcinoma (HNSCC) that originates primarily from the palatine and lingual tonsils or the mucosal lining of the throat and soft palate. It is a significant global health burden due to its rising incidence rates and impact on patients’ quality of life (1–3). OPSCC is strongly associated with certain risk factors, such as tobacco and alcohol abuse and infection with high-risk strains of the human papillomavirus (HPV) (4).

In recent years, there has been a growing recognition of the need for innovative approaches to improve OPSCC patient outcome. Traditional treatment modalities, including surgery, radiation, and chemotherapy, have shown limitations and adverse effects (5–8). Adoptive cell transfer (ACT) and peptide vaccines have presented promising results in treating head and neck cancers (9–18). Overexpressed self-proteins in OPSCC are relevant in targeted immunotherapies because they drive tumorigenesis and disease progression. One such overexpressed tumor associated protein is p53, a tumor suppressor protein whose dysregulation or mutation is frequently observed in HNSCC and contributes to the accumulation of genetic abnormalities and uncontrolled cell growth (19). Another essential protein is the epidermal growth factor receptor (EGFR), a cell surface receptor that regulates cell proliferation, survival, and metastasis. Overexpression and amplification of EGFR are commonly observed in OPSCC and associated with poor prognosis and resistance to treatment (20). Additionally, alterations in cell cycle proteins, such as cyclins and cyclin-dependent kinases (CDK), have been implicated in the development and progression of HNSCC (21). Cancer testis antigens (CTA) represent a unique class of tumor-associated antigens that are predominantly expressed in testicular germ cells under normal conditions but are aberrantly and ectopically expressed in various malignancies, including HNSCC, providing an ideal target for immunotherapeutic interventions. This restricted expression profile limits the potential for off-target effects, making CTA particularly attractive for cancer-specific targeting (22, 23). Furthermore, emerging evidence suggests many CTA play active roles in cancer progression and their expression is not merely an incidental outcome (23). For instance, CTA such as NY-ESO-1, FTHL17, and SPATA19 are essential for tumor cell proliferation and survival (24). Additionally, many CTA inhibit apoptosis and promote chemotherapeutic resistance, often through interactions with p53 (25–28). CTA like CT45A1, OIP5, and CAGE have been shown to enhance invasion, metastasis, and angiogenesis (29–32). Some CTA also influence cellular energetics by promoting glycolysis and mitochondrial function (33–36). Furthermore, CTA such as HORMAD1 contribute to genome instability by disrupting DNA repair mechanisms, which increases sensitivity to certain chemotherapeutics (37). Studies have also shown that elevated mRNA levels and protein expression of CTA, such as those from the melanoma antigen-A (MAGE) family and NY-ESO-1, along with specific CTA antibody responses are associated with poor survival outcomes in HNSCC patients (38–40). Additionally, the expression of CTA and the pattern of antibody responses have been shown to vary significantly with HPV status, indicating a differential immunogenic landscape in HPV+ versus HPV- patients (41, 42). Similarly, recent findings demonstrated that the humoral immune responses against tumor-associated antigens in HNSCC patients are influenced by HPV status and tumor stage (43). Clinical investigations into CTA-based vaccines have demonstrated their immunogenicity, albeit with varied clinical outcomes. For instance, a study involving a MAGEA3 peptide vaccine indicated the induction of specific T cell responses in both tumor infiltrating lymphocytes and peripheral blood mononuclear cells, with acceptable toxicity levels (11). Similarly, in a phase 2 clinical trial, a NY-ESO-1 vaccine combined with adjuvant therapy elicited strong CD4+ and CD8+ T cell responses, indicating a readily amplifiable functional T cell repertoire (44). In another clinical trial, CD4+ and CD8+ T cells specific to NY-ESO-1 were successfully induced in 57% of sarcoma patients, correlating with improved survival rates (45). Another phase 2 clinical trial conducted in advanced esophageal cancer patients and evaluating the impact of three HLA restricted CTA peptides TTK, LY6K, and IMP3 showed that the progression free survival was significantly better in patients positive for required HLA haplotype and correlated to peptide-specific T cell responses (46, 47). Additionally, SSX2-specific antibody responses have been detected in the serum of HNSCC patients, confirming the immunogenicity of this CTA (48). The distinctive expression pattern of CTA, combined with their demonstrated capability to evoke robust immune responses, makes them particularly promising candidates for developing targeted immunotherapies that could offer more effective and less toxic treatment options for OPSCC patients.

Gene and protein expression analyses utilized to outline HNSCC immunotherapy targets (49, 50) help identify overexpressed tumor-associated antigens. However, they do not directly reflect their presentation on the HLA molecules. Post-transcriptional and post-translational modifications and other complex regulatory mechanisms can contribute to discrepancies among gene expression, protein production, and ligand presentation on HLA molecules (51, 52). Additionally, the availability and efficiency of antigen processing and presentation machinery can influence the display of antigens (53, 54), further impacting the efficacy of using gene or protein expression alone to identify immunotherapy candidates. Ligand presentation on HLA molecules is crucial for immune recognition and response. Assessing the presentation of antigens on HLA molecules provides valuable insights into the repertoire of tumor-associated peptides (TAP) recognized by the immune system. In addition, antibody response (AR) profiling allows the assessment of humoral immune responses directed against TAP (41, 42).

Previous studies in OPSCC have primarily focused on individual omics layers to identify overexpressed proteins for immunotherapy targets, potentially overlooking valuable information from other layers, such as ligand presentation on HLA molecules and antibody response (AR), which play important roles in the success of vaccine or ACT-based immunotherapies. Therefore, this study aimed to evaluate the correlation between tumor-associated peptide (TAP) presentation on HLA molecules and gene expression in OPSCC patients, and to determine whether clinical parameters such as HPV status and tumor stage influence this correlation. Additionally, we sought to identify TAP uniquely presented on HLA class I and II in tumors (TAP-U) by comparing them against a comprehensive healthy tissue atlas, and to assess the concordance between TAP-U presentation on HLA class II and their respective humoral response.

## 2 Materials and methods

### 2.1 Patient samples

This study cohort consists of 40 OPSCC patients diagnosed and treated at the University Hospital Ulm, Germany. Patients were selected based on the resectability of their tumors and balanced clinical parameters, such as tumor stage and HPV status, across the subgroups to ensure a comprehensive and representative sample population. Representative tissue samples from 40 untreated patients were retrieved from the primary tumor site. HPV status was determined by RNA-seq. Matched blood samples were collected from 26 of the untreated patients using serum-separating tubes, which were then centrifuged at 2500 xg to separate and aliquot the serum. This method was chosen to ensure high-quality serum samples for subsequent analysis. In one case, a blood sample was collected in a citrate tube and subjected to a two-step centrifugation process (800 xg followed by 2500 xg for 15 and 10 minutes, respectively) to obtain plasma. Both plasma and serum aliquots were stored at -20°C to preserve their integrity until analysis. All samples were taken from resection specimens without impairing the readability of pathological assessments. No additional biopsies or invasive procedures were required, thus minimizing any potential risk or discomfort to the patients. Informed consent was obtained from all participants in accordance with the principles outlined in the Declaration of Helsinki. The study was conducted following the guidelines established by the local ethics committee (approval numbers 222/13 and 90/15).

### 2.2 RNA sequencing

Snap-frozen tumor tissues were used to extract total RNA using the AllPrep DNA/RNA Mini Kit (Qiagen, Germany). The Agilent 2100 Bioanalyzer Instrument was used to measure total RNA and assess its quality (Agilent RNA 6000 Pico). TruSeq Stranded mRNA was used to create libraries from 500 ng of input material according to manufacturer’s instructions. Agilent 2100 Bioanalyzer Instrument was then used to quantify and check the libraries’ quality (DNA 7500 kit). In one lane of the HiSeq 2500/NovaSeq 6000 System, libraries were pooled and single-end sequenced. Using bcl2fastq, sequence data was converted to FASTQ format (2.20.0.422). High-quality reads were transformed to gene-specific read counts using featureCounts after being mapped to the human genome (hg38) using STAR (2.0.9) and removing multimapping reads (2.0.0). Using a viGen bioinformatic workflow, unmapped reads were aligned to HPV high-risk type genomes. Samples were deemed HPV-positive if they had at least 500 reads for the HPV E6 or E7 RNA or at least 500 reads for all HPV oncogenes combined (E1, E2, E4, E5, E6, E7, L1, L2).

### 2.3 Whole exome sequencing and variant calling

50 mL of whole blood were collected in citrate tubes. Peripheral blood mononuclear cells (PBMC) were isolated using density gradient centrifugation with Leucosep tubes according to the manufacturer’s protocol (Greiner Bio-One, Germany). Approximately 5 × 10^6 PBMC were aliquoted and preserved in RPMI1640 medium supplemented with fetal bovine serum and 10% dimethyl sulfoxide, then stored at -80°C until further use. DNA was extracted from the matching tumor and PBMC samples, used as healthy references, using the Qiagen AllPrep DNA/RNA Mini Kit. Agilent 4200 TapeStation System (Agilent Genomic DNA ScreenTape) was used to assess the quality of total DNA, and Quant-iTTM PicoGreenTM was used to quantify it. SureSelect Human All Exon V6 was used to create libraries from 3 g of input material according to manufacturer’s instructions. Agilent 4200 TapeStation System was then used to quantify and check the libraries’ quality (D1000 ScreenTape). On a NextSeq 500 System (High Output Flow Cell) in 150 cycles (2x 75bp paired end) mode, libraries were pooled and sequenced. Bcl2fastq v2.20.0.422 was used to convert sequence data to FASTQ format. High quality reads were aligned to the hg38 reference genome and VCF files were generated using strelka (2.9.10).

### 2.4 Ligandome isolation and analysis

As we previously described (55), standard immunoaffinity purification was used to extract HLA class I- and II-presented peptides from tissue samples and eluted peptides were examined using tandem mass spectrometry (MS/MS) in a Thermo Fisher Scientific on-line coupled LTQ Orbitrap XL hybrid mass spectrometer that has a nano-electron spray ion source. Using Proteome Discoverer 1.4, MS data processing was conducted. 49 different HLA class I allotypes were detected in our cohort, accounting for at least 1 HLA class I allotype in 99.93% of the world’s population. The cohort included 33 different HLA-DRB, 13 different HLA-DQB, and 12 different HLA-DPB allotypes, accounting for at least 1 HLA class II allotype in 99.99% of people worldwide.

### 2.5 Antibody expression analysis

Autoantibodies against 262 tumor-associated antigens (comprising 213 CTA; 39 tumor-associated antigens; and 10 variant forms of p53; **Supplementary Table 1**) were measured in 27 serum/plasma samples stored in the Biobank. The measurement of antibody expression was conducted with the KREX™ functional proteomics CT262 array (Sengenics, Singapore). Protein/antibody binding was quantitatively assessed using the G4600AD Microarray Scanner, ensuring high-resolution detection of the interactions. Image acquisition and analysis were conducted using GenePix Pro 7 software (Molecular Devices). Data was normalized using positive control proteins on the array (**Supplementary Table 1**).

### 2.6 Data analysis and statistics

**Figure 1:**
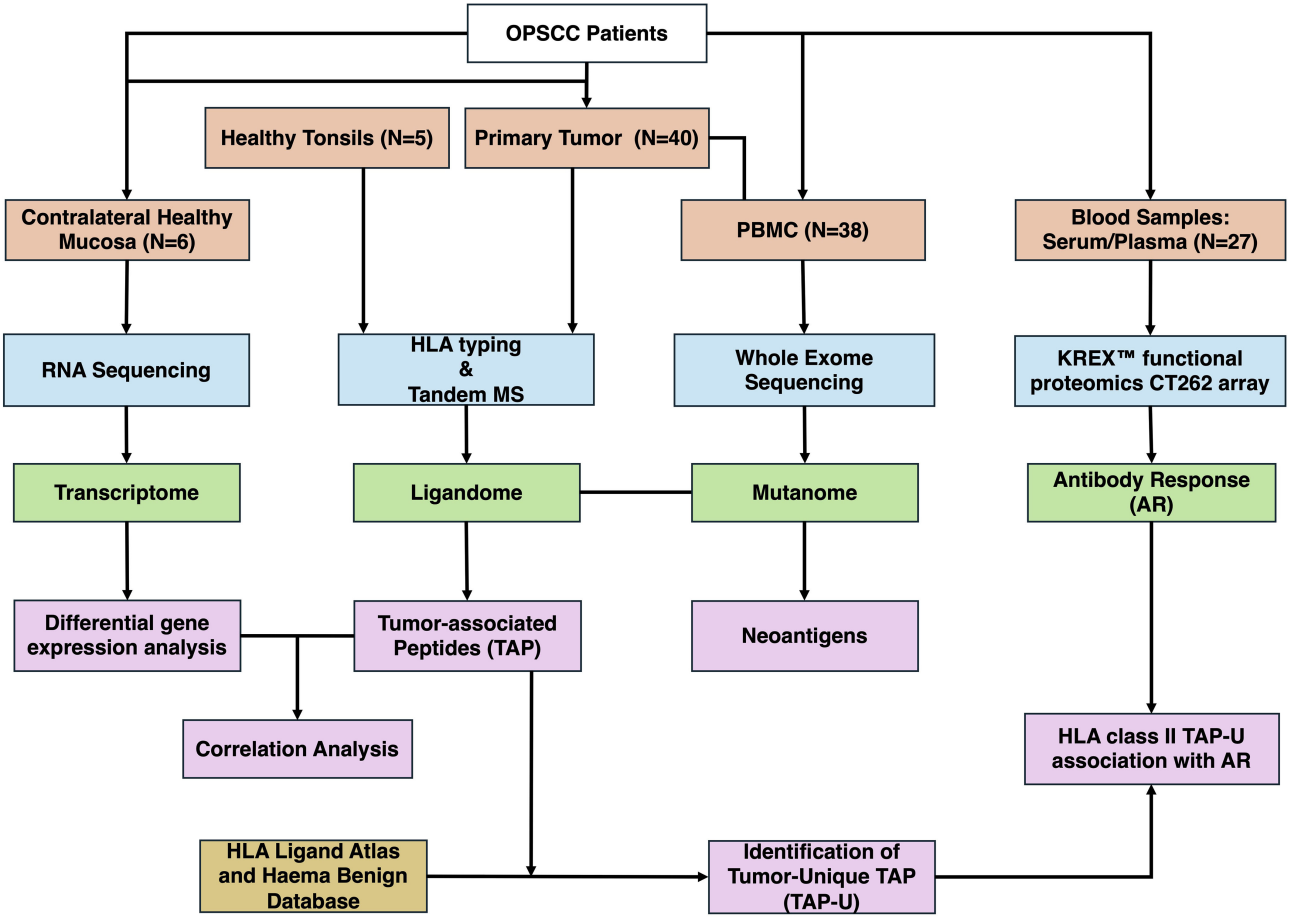
Overview of the study workflow. OPSCC, Oropharyngeal Squamous Cell Carcinoma; PBMC, Peripheral Blood Mononuclear Cells; MS, Mass Spectrometry; AR, Antibody Response; TAP, Tumor-associated Peptides; TAP-U, Tumor-unique TAP.

(**Figure 1**) illustrates the analysis process of the study. Data analysis was performed in R (4.1.1). Data wrangling was carried out utilizing tidyverse (1.3.1), data.table (1.14.2), and dplyr (1.0.9) and focused on 312 genes (**Supplementary Table 2**). Differential expression analysis was performed using deseq2 (1.34.0), for its ability to handle variance in data and incorporate shrinkage estimators to improve the reliability of log fold changes. We employed the “apeglm” shrinkage estimator to stabilize the variance of log fold changes and prevent the exaggeration of fold changes for genes with low counts. The threshold for differentially expressed genes was determined with an absolute log fold change (LFC) > 1 and a false discovery rate (FDR) < 0.05. The RNA expression transcript per million (TPM) values were used for correlation. Correlation matrices were generated using mixOmics (6.19.4). The point-biserial correlation, calculated using the base stats package in R, was used to assess the relationship between continuous (TPM or Antibody levels) and binary (semi-quantitative MS data) variables, assuming a linear relationship. Correlations with p-value < 0.05 were considered significant. Venn diagrams were generated using ggvenn (0.1.9). The prediction of peptide binding to MHC class I types A, B, and C was performed using NetMHCpan (4.0). Epitopes were identified as high-affinity binders if they showed a predicted binding affinity of 500 nM or less. Sankey plot for tumor unique ligands (TAP-U) was generated utilizing networkD3 (0.4). Survival analysis was conducted by fitting survival curves using the survival package (3.3–1) and generating Kaplan-Meier plots utilizing the survminer package (0.4.9). The immunedeconv (2.0.4) R wrapper package was used to perform immune deconvolution on RNA-Seq data using the quanTIseq program as method. KEGG pathway enrichment analysis was conducted using clusterProfiler (4.10.1) and org.Hs.eg.db (3.18.0), pathways with p-value <0.05 and q-value < 0.1 were considered significant. Oncoplot was produced using maftools (2.10.05). Visualization was done using ggplot2 (3.3.6).

## 3 Results

### 3.1 Cohort characteristics

**Table 1** details the clinicopathological features of the OPSCC patients (n = 40). The mean age at the diagnosis was 59.93 years, ranging from 38.17 to 79.33 years. 82.5% of the patients were men. The median range of smoking in pack years was 24.8. 52.5% of patients were stage I or II, and 47.5% were stage III or IV, according to the 8th edition of the Union for International Cancer Control (UICC). No distant metastases were reported. The human papillomavirus (HPV) status was positive in 22 patients. 15 patients had a recurrence.

**Table 1:** Clinicopathological characteristics of the OPSCC cohort in this study.

Patient characteristics			
Characteristics	Variables		
		n	%
**Sex**	Male	33	82,5%
	Female	7	17,5%
**Age**	Mean (range)	59.93 (38.17-79.33)	
**Smoking (Pack years)**	Median (range)	24.8 (0-60)	
**Stage (UICC v8)**	I	8	20%
	II	13	32.5%
	III	9	22.5%
	IV	10	25%
**HPV-status**	Negative	18	45,0%
	Positive	22	55,0%
**Recurrence**	Yes	15	37,5%
	No	25	62,5%

### 3.2 Comparative profiling of tumor-associated peptides presentation on HLA molecules

**Figure 2:**
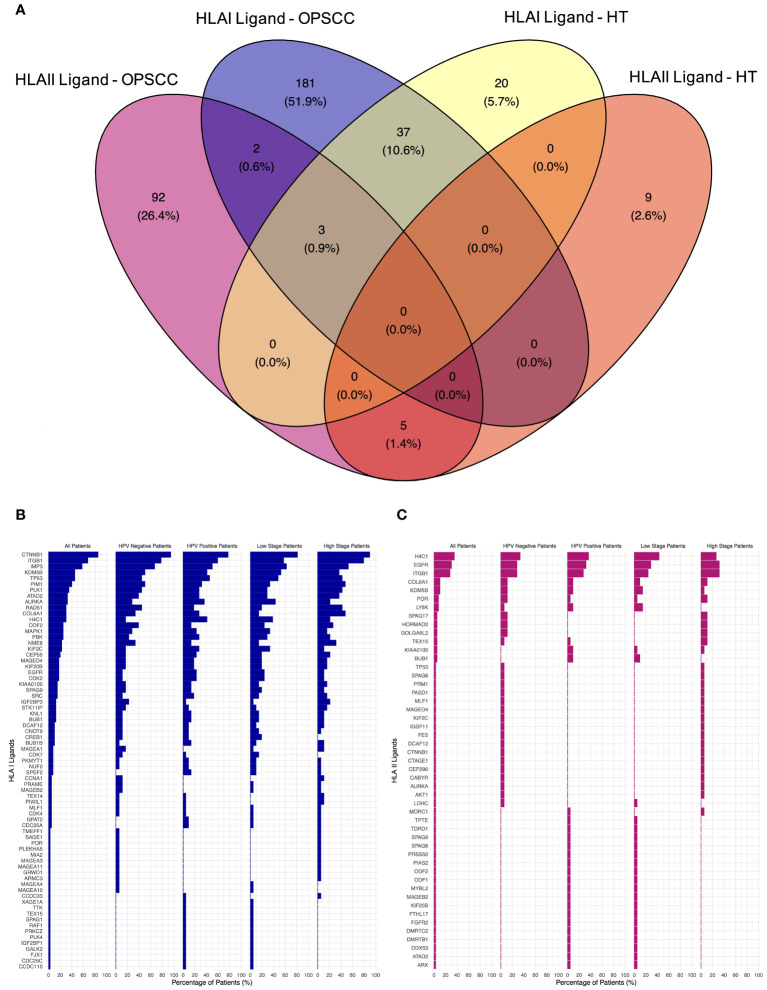
Comparative profiling of the OPSCC ligandome. **(A)** Venn diagram illustrating the overlap between HLA class I and II ligands in OPSCC and healthy tonsils (HT). 183 HLA class I and 94 HLA class II tumor-associated peptides (TAP) were outlined. **(B, C)** Waterfall plots of TAP source genes of HLA class I **(B)** and class II **(C)** showing the percentage (%) of OPSCC all, HPV+, HPV-, low-stage, and high-stage patients with presentation.

The search for neoantigens carrying mutated sequences in the ligandome was conducted. The top five mutated genes in our TAP list were Tumor Protein P53 (*TP53*), Epidermal Growth Factor Receptor (*EGFR*), Kinetochore Scaffold 1 (*KNL1*), Testis expressed 15, meiosis and synapsis associated (*TEX15*), and Sperm associated antigen 17 (*SPAG17*). 16% of the patients revealed no mutations for the genes filtered (**Supplementary Figure 1**). No peptides carrying mutation motifs for any of the 211 mutated genes in our cohort could be detected in the ligandome, as we previously reported (55). A total of 223 HLA class I and 102 HLA class II presented peptides were discovered by LC-MS/MS to map back to a list of 312 genes (**Supplementary Table 2**). Comparative profiling of the HLA-bound peptides that were isolated from tumor and healthy tonsils (HT) was done to determine tumor-associated peptides (TAP) (**Figure 2A**). 40 of HLA class I ligands from HT and tumors overlapped. 183 TAP were identified on HLA class I. The top 5 HLA class I TAP source proteins as per presentation ratio in our cohort were Catenin beta-1 (CTNNB1), Integrin Subunit Beta 1 (ITGB1), IMP U3 Small Nucleolar Ribonucleoprotein 3 (IMP3), TP53, and Lysine-specific demethylase 5B (KDMB5), respectively. Stratification based on HPV status and tumor stage, revealed that the top five TAP source genes consistently exhibited the highest presentation ratios across all subpopulations (**Figure 2B**). Regarding HLA class II ligands, 8 peptides from HT (5 on HLA class II and 3 on HLA class I) and tumors overlapped. 94 TAP were identified (**Figure 2A**). The top 5 HLA class II TAP genes were H4 Clustered Histone 1 (H4C1), EGFR, ITGB1, KDM5B, and Collagen Type VI Alpha 1 Chain (COL6A1), respectively. Notably, H4C1, EGFR, and ITGB1 maintained their prominence in terms of presentation levels across subpopulations stratified by HPV status and tumor stage (**Figure 2C**).

### 3.3 Tenuous concordance between TAP presentation and gene expression levels

**Figure 3:**
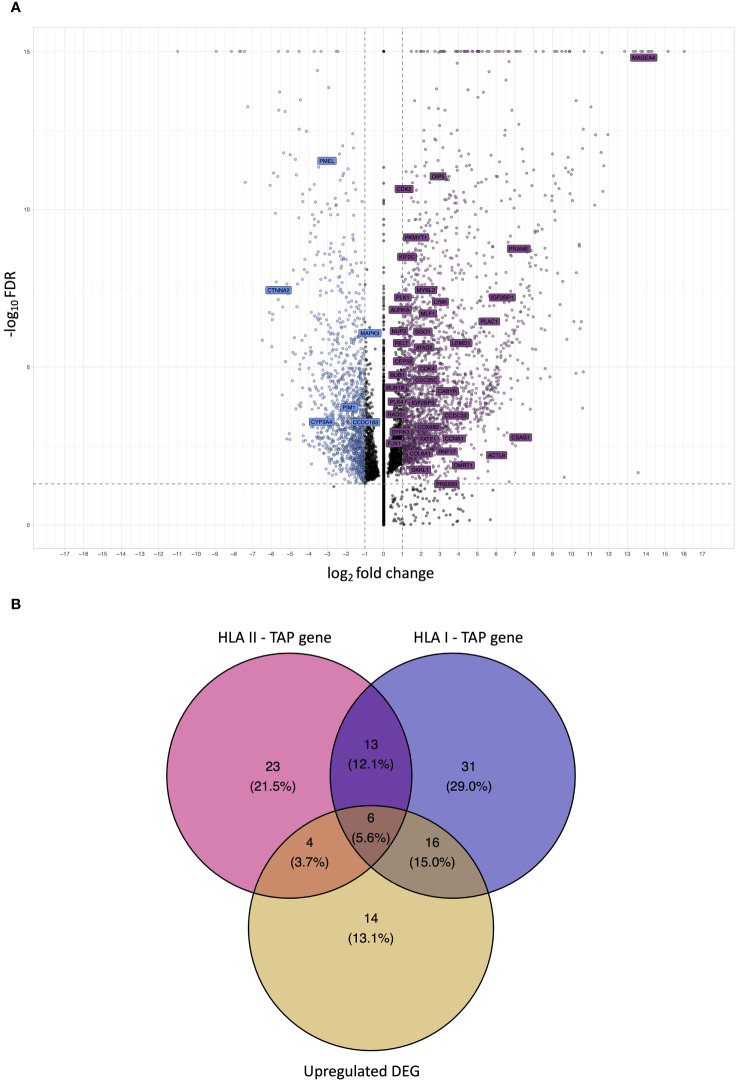
Analysis of differential gene expression to determine upregulated differentially expressed genes (DEG) and intersection with TAP. **(A)** A volcano plot displaying differential gene expression (log2foldchange – LFC) between OPSCC tumors and contralateral healthy mucosa. Each dot represents one gene. Genes depicted in violet are upregulated DEG in the tumor, whereas genes depicted in blue are downregulated. Labelled genes are the source of the outlined OPSCC TAP. Gray dotted lines represent cutoff thresholds for 0.05 FDR and an absolute LFC of 1. **(B)** Venn diagram depicting the intersection among HLA class I TAP genes, HLA class II TAP genes, and upregulated DEG. 22 HLA class I and 10 HLA class II TAP intersected with upregulated DEG, respectively.

**Figure 4:**
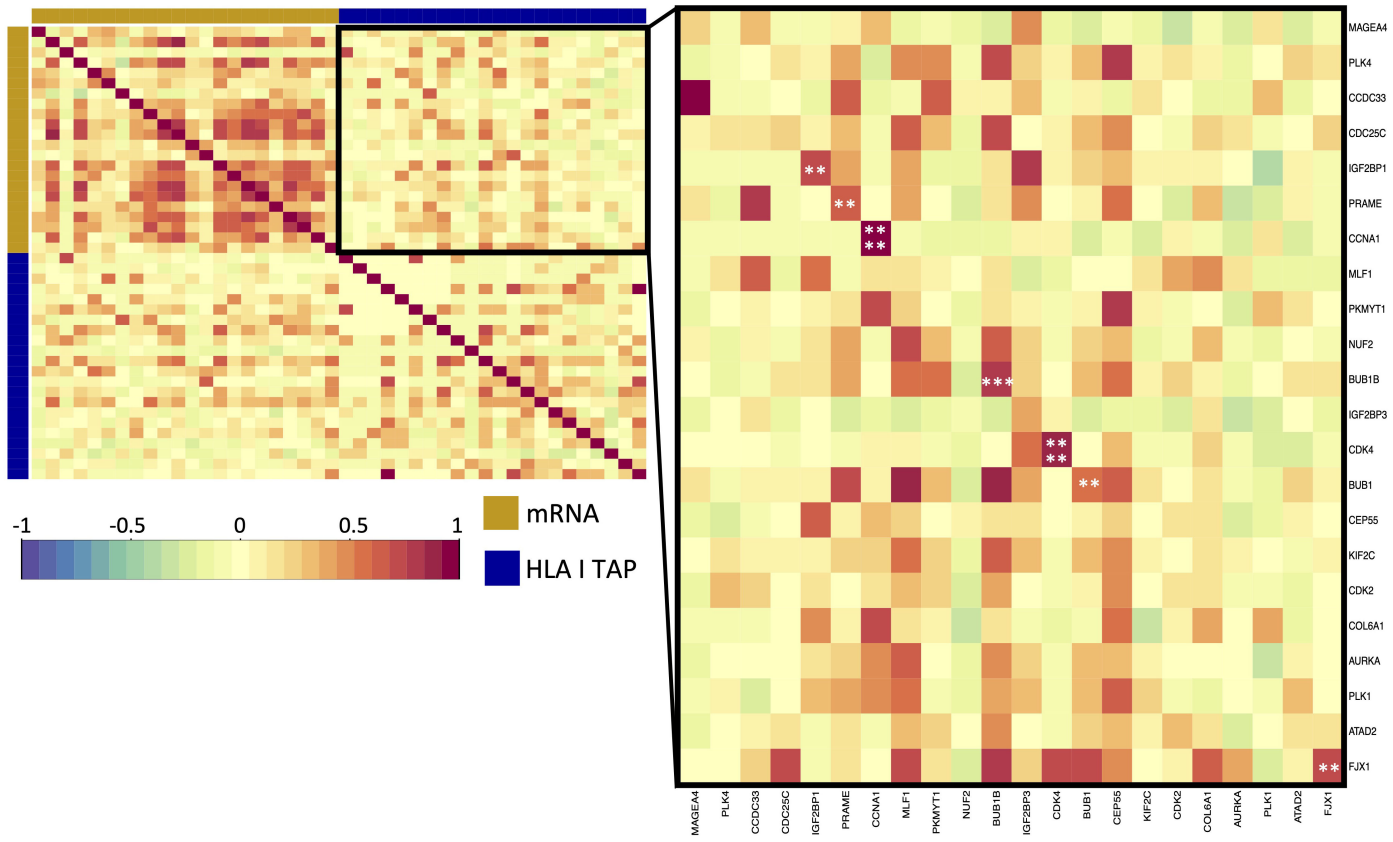
Correlation of upregulated HLA class I TAP genes with their level of presentation on HLA class I. Correlation matrix between gene expression and HLA class I TAP presentation. Intercorrelation coefficients between the two datasets were obtained using the point-biserial correlation. Red and blue represent positive and negative correlations, respectively. The yellow sidebar indicates the expression dataset and indigo indicates the HLA class I TAP dataset. **, ***, **** correspond to p ≤ 0.01, p ≤ 0.001, p ≤ 0.0001, respectively.

**Figure 5:**
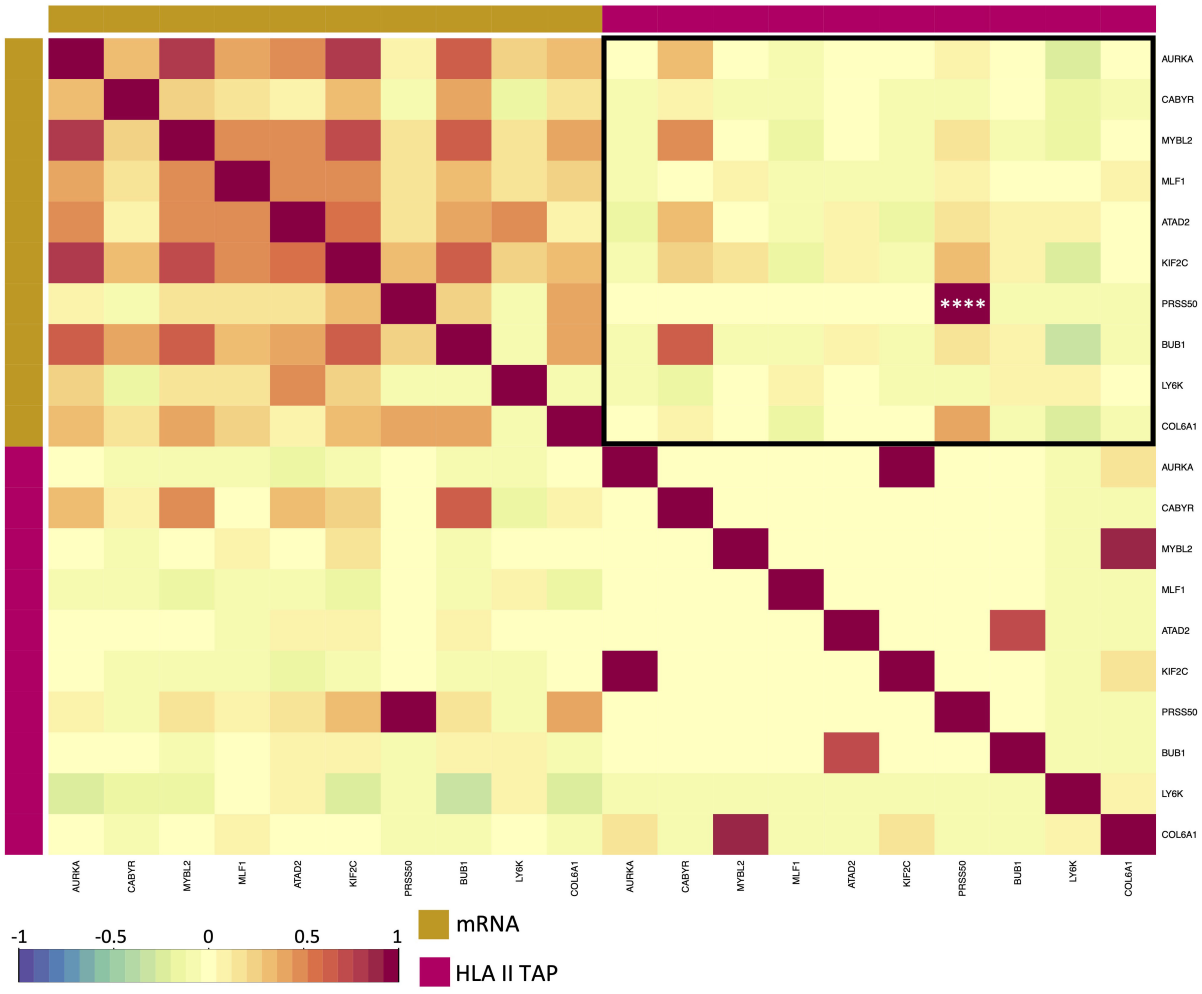
Correlation between elevated HLA class II TAP gene expression and their presentation level on HLA. Correlation matrix between expression and HLA class II TAP presentation; red and blue represent positive and negative correlations, respectively. The yellow sidebar indicates the expression dataset and pink indicates the HLA class II TAP dataset. **** correspond to p ≤ 0.0001.

Differential gene expression analysis was conducted to outline the differentially expressed genes (DEG) between the tumor and contralateral healthy mucosa. Significant expression differences between tumor and healthy tissue were found for 46 genes from our curated list, 40 of which were upregulated in tumors, and 6 were downregulated (**Figure 3A**, **Supplementary Table 3**). Interestingly, mapping the TAP back to their respective genes, only 26 of the TAP genes were shown to have an upregulated level of expression in tumors in comparison to healthy tissue. 16 of those genes exhibited the presentation of respective ligands only on HLA class I, 4 were presented only on HLA class II, and 6 were presented on both HLA classes (**Figure 3B**). Consequently, to better understand how our TAP genes’ expression and immunopeptidome profiles correlate, we combined MS and RNAseq data via N-integration. Correlation analysis was done by integrating ligands from one HLA class at a time. A weak positive correlation (r = 0.25, p < 0.05) between TAP presentation on HLA class I and the level of respective mRNA in tumors of patients was observed (**Figure 4**). Seven TAP genes, five of which are CTA, had a relatively stronger correlation (r > 0.4, p < 0.05), taken individually, Melanoma antigen preferentially expressed in tumors (*PRAME*), Insulin-like growth factor 2 mRNA-binding protein 1 (*IGF2BP1*), BUB1 mitotic checkpoint serine/threonine kinase (*BUB1*), BUB1 mitotic checkpoint serine/threonine kinase B (*BUB1B*), Cyclin-A1 (*CCNA1*), Cyclin-dependent kinase 4 (*CDK4*), and Four-jointed box protein 1 (*FJX1*). No holistic correlation was found between the TAP presentation on HLA class II and gene expression level. Probable Serine protease 50 (*PRSS50*), however, recorded a robust correlation (r = 0.99, p < 0.05) (**Figure 5**). Patients were then stratified based on HPV status, yet the overall correlation did not significantly change. However, the stratification emphasized that the correlation pattern for some of the latter genes was indeed contingent upon HPV status. *IGF2BP1* and *FJX1* exclusively correlated in HPV+ patients, while *PRAME*, *BUB1B*, *CCNA1*, *CDK4*, and Insulin-Like Growth Factor 2 mRNA Binding Protein 3 (*IGF2BP3*) in HPV- patients. In our cohort *CCNA1* was exclusively presented in HPV- tumors. Additionally, *IGF2BP3* also demonstrated a higher level of ligand presentation in HPV- compared to HPV+ patients (**Figure 2B**). Stratification based on the UICC stage showed that *IGF2BP1*, *FJX1*, and *MAGEA4* correlated exclusively in the low stage, while *BUB1B*, *CCNA1*, *CDK4*, and *IGF2BP3* in high-stage patients. CCNA1 was observed to be exclusively presented on HLA class I in patients with high stage tumors (**Figure 2B**). *PRSS50* presentation on HLA class II and gene expression exclusively correlated in HPV+ and low-stage patients (**Supplementary Table 4**).

### 3.4 Identification of tumor-unique TAP as potential immunotherapy targets

**Figure 6:**
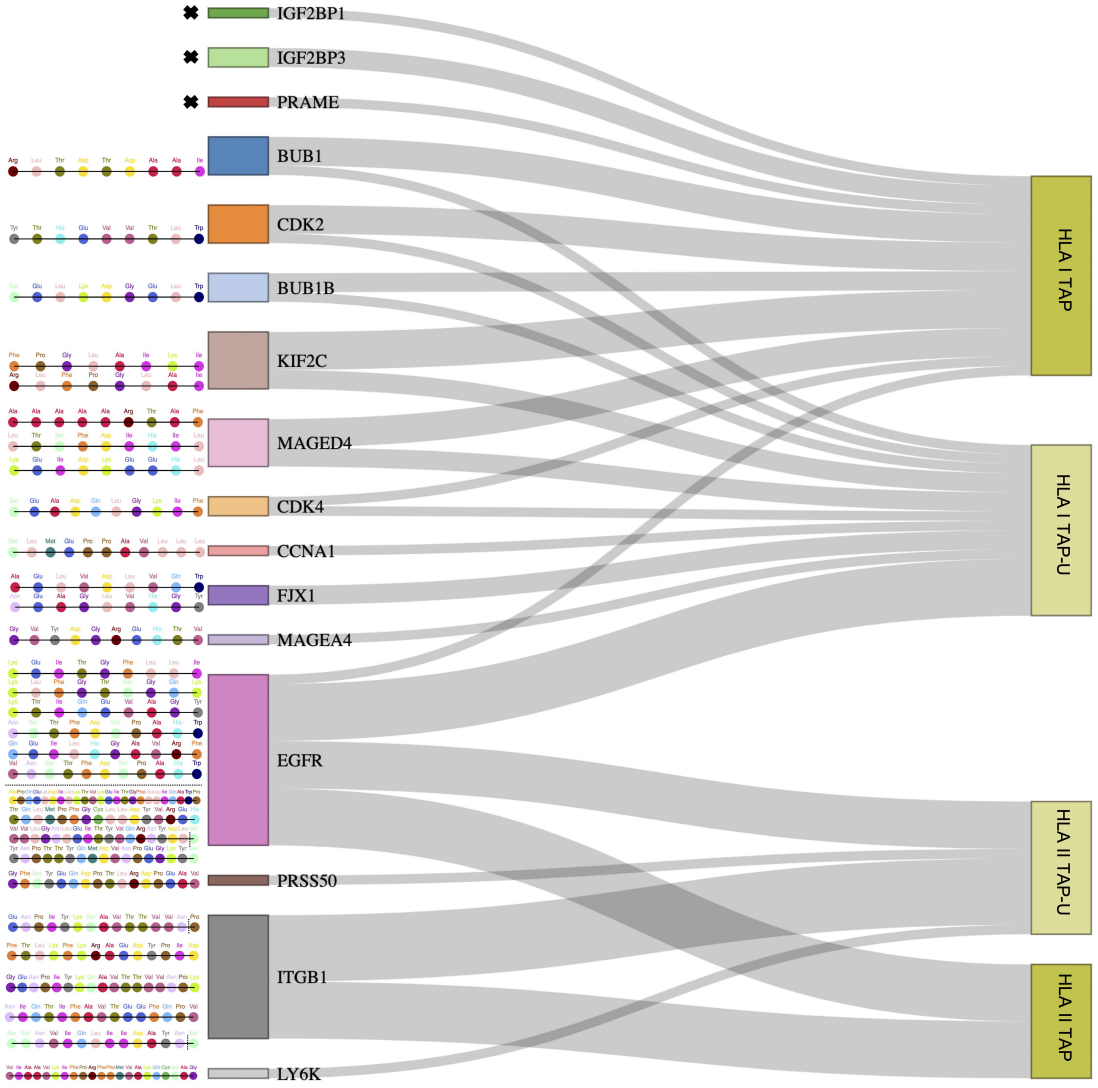
Outlining tumor-unique peptides (TAP-U) of positively correlated and most presented genes. Sankey diagram depicting gene-derived ligand classification into TAP and TAP-U categories. The flow of genes towards each category represents the number of peptides mapping to each category. The respective peptide amino acid sequences of TAP-U genes are detailed on the left side. Amino acids with dotted vertical lines represent shorter peptide variants.

**Figure 7:**
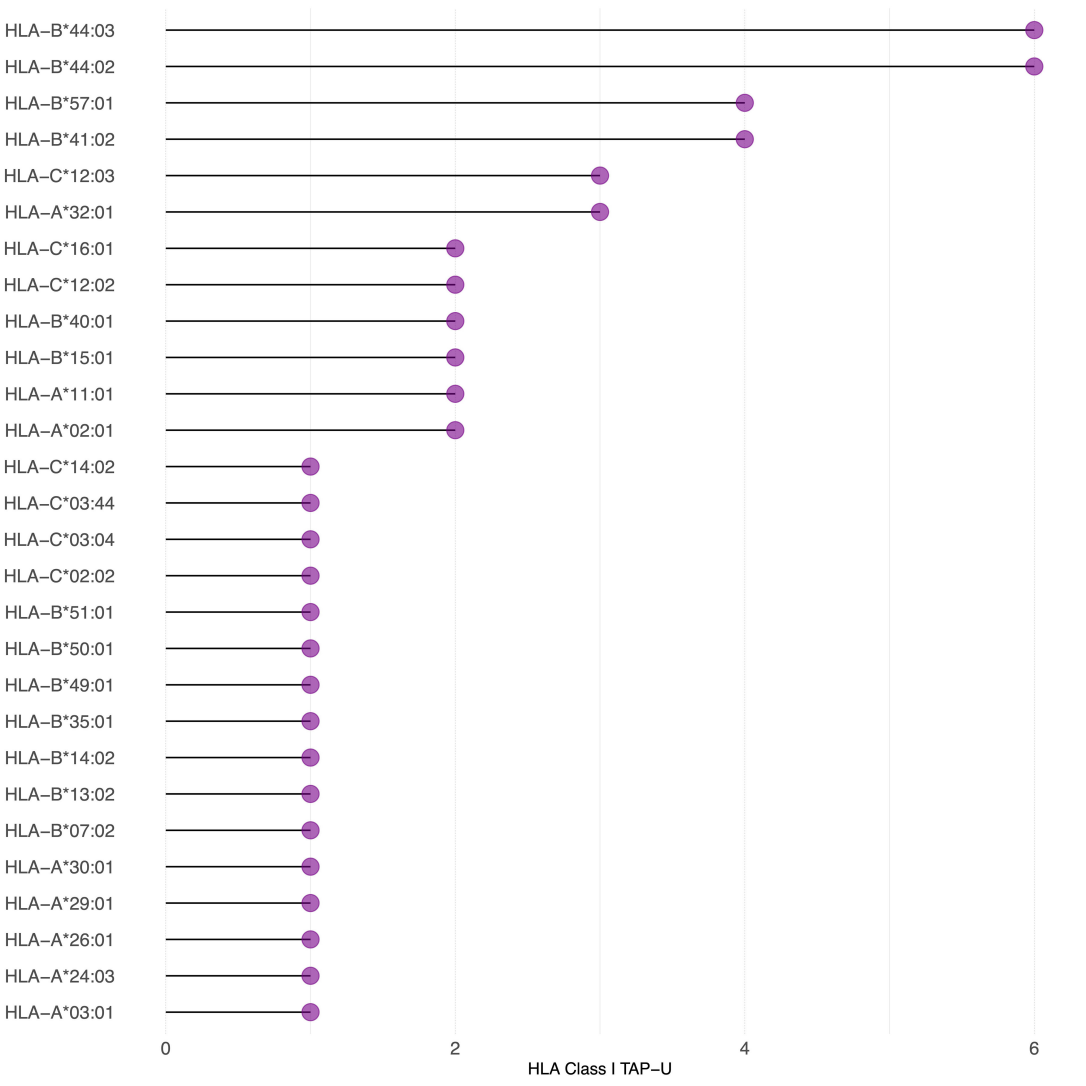
TAP-U restricted by HLA class I haplotypes identified in the cohort. This waterfall plot illustrates the number of TAP-U peptides restricted on the x-axis and the corresponding HLA class I haplotypes on the y-axis. Restriction was considered for binding affinity ≤ 500 nM.

Since we observed that only 26 TAP genes were upregulated, among which seven showed a positive correlation, we expanded our analysis to include all TAP, irrespective of their gene regulation status. We then conducted a comprehensive filtering process against peptides presented on 30 different benign tissues, besides HT, obtained from the HLA Ligand Atlas and Haema benign database. 73 HLA class I TAP were shown to be unique for the tumor (TAP-U) and were not presented in any of the benign tissue. HLA class I TAP-U mapped back to 46 genes, including seven of the genes that showed a positive correlation between expression and presentation, namely *BUB1*, *BUB1B*, *CDK2*, *CDK4*, *FJX1*, *CCNA1* and *MAGEA4*. According to the presentation ratio among patients, the top 3 presented HLA class I TAP-U were derived from the Kinesin Family Member 2C (*KIF2C*), Melanoma-associated antigen D4 (*MAGED4*), and *EGFR*. 62 HLA class II TAP were considered unique and belonged to 44 source genes, including *PRSS50*. The expression of Lymphocyte Antigen 6 Family Member K (*LY6K*), *EGFR*, and Integrin Subunit Beta 1 (*ITGB1*) was the source of the top 3 most presented HLA class II TAP-U, (**Figure 6**, **Supplementary Table 5**). Predictive analysis of the latter HLA class I TAP-U restriction identified the common and highly prevalent HLA-B44:03 and HLA-B44:02 as the haplotypes with the highest number of TAP-U restricted peptides, primarily sourced from the BUB1B, EGFR, CDK4, and FJX1 proteins. Additionally, HLA-B57:01, HLA-B41:02, HLA-C12:03, and HLA-A32:01 also demonstrated significant capabilities for restricting TAP-U peptides, particularly those derived from CDK2, EGFR, MAGED4, CDK4, and KIF2C (**Figure 7**, **Supplementary Table 6**). We then performed a log-rank test to evaluate the impact of the top 3 TAP-U presentations on disease-free survival (DFS) in patients, specifically investigated differences in DFS between patient groups categorized by the presence or absence of these TAP-U presentations. A significant difference (p < 0.05) was observed in the DFS curves, particularly associated with the presentation of the EGFR peptide KTIQEVAGY on HLA class I. All patients presenting this peptide had OPSCC recurrence. Conversely, for the top three HLA class II TAP-U, no significant differences in DFS were detected. However, an LY6K peptide, VIAAVKIFPRFFMVAKQCSAG, which was exclusively presented in patients with low-stage tumors, was found to be prognostic, but only within this group (**Supplementary Figure 2**).

### 3.5 Analysis of antibody response to HLA class II TAP-U

**Figure 8:**
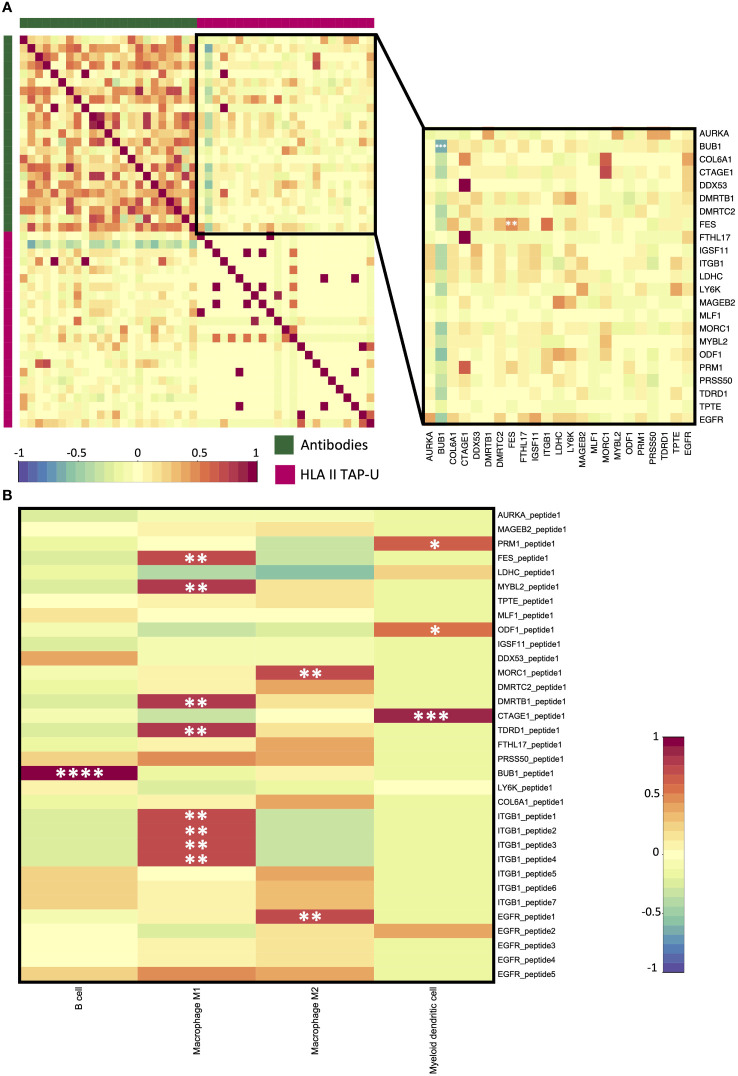
HLA class II TAP-U antibody response (AR) and immune infiltration. **(A)** A correlation matrix between AR and HLA class II TAP-U presentation datasets. Intercorrelation coefficients between the two datasets were obtained using the point-biserial correlation. The green sidebar indicates the antibody dataset and pink indicates the HLA class II TAP-U dataset. **(B)** A correlation matrix between positively correlated HLA class II TAP-U and computed infiltration ratios of professional antigen presenting cells (APC). Red and blue represent positive and negative correlations, respectively. *, **, ***, **** correspond to p ≤ 0.05, p ≤ 0.01, p ≤ 0.001, p ≤ 0.0001, respectively.

Next, we aimed to examine antibody response (AR) patterns to HLA class II TAP-U. 33 HLA class II TAP-U belonging to 23 genes had variance across the cohort and were among the antibodies detected by the KREX™ array. Our results indicated no substantial quantitative correlation between AR levels and almost all HLA class II TAP-U presentation. A notable exception was observed for the FES proto-oncogene tyrosine kinase (FES), which showed a significant positive correlation (r = 0.51, p < 0.05). On the other hand, BUB1 emerged as the sole TAP-U demonstrating a negative correlation with its antibody response (r = -0.60, p < 0.05). This negative correlation was also evident with the antibody levels against most of the other antigens (**Figure 8A**). To further understand exclusive antigen presentation dynamics, we explored the associations between the presentation levels of the 33 HLA class II TAP-U and the computed ratios of various antigen-presenting cells (APC) – specifically B cells, macrophages, and myeloid dendritic cells. This investigation aimed to identify any unique associations of these HLA class II TAP-U with specific APC subsets. We discovered that peptides from ITGB1, FES, MYB Proto-Oncogene Like 2 (MYBL2), DMRT Like Family B With Proline-Rich C-Terminal 1 (DMRTB1), and Tudor Domain Containing 1 (TDRD1) were predominantly presented in association with high M1 macrophage ratios within tumors. In contrast, two peptides derived from MORC Family CW-Type Zinc Finger 1 (MORC1) and EGFR were linked to M2 macrophage infiltrates. Additionally, ligands from Protamine 1 (PRM1), Outer Dense Fiber of Sperm Tails 1 (ODF1), and Cutaneous T Cell Lymphoma-Associated Antigen 1 (CTAGE1) demonstrated an exclusive correlation with myeloid dendritic cell infiltration levels. Notably, the BUB1 peptide was the only ligand that showed a positive correlation with the infiltration level of B cells (**Figure 8B**). KEGG pathway analysis of proteins from which the correlating peptides were derived showed significant enrichment in pathways that are known to influence cellular migration, adhesion, and immune cell interactions, such as focal adhesion, and regulation of actin cytoskeleton. Other enriched pathways included proteoglycans in cancer, ECM-receptor interaction, Rap1 signaling pathway, and PI3K-Akt signaling pathway. Pathways typically associated with infections like shigellosis, pertussis, and leishmaniasis, which include mechanisms related to antigen uptake and presentation, were also noted. Significant was the enrichment of the PD-L1 expression and PD-1 checkpoint pathway in cancer, as well as the HPV infection pathway, which may have implications for antigen presentation dynamics in OPSCC (**Supplementary Figure 3**).

## 4 Discussion

The field of tumor immunotherapy, which utilizes the immune system to combat malignant tumors, encompasses both passive and active approaches (56). Active immunotherapy primarily involves vaccination and adoptive cell transfer (ACT) (57). Many OPSCC immunotherapy targets, including cancer-testis antigens (CTA), emerged and were considered because of their differential mode of expression. This study set out to bridge the gap and address the limitations in OPSCC research by evaluating the correlation between tumor-associated peptide (TAP) presentation on HLA molecules and gene expression, considering clinical parameters such as HPV status and tumor stage. Additionally, this study aimed to identify uniquely presented TAP on HLA class I and II in tumors (TAP-U) and assess HLA class II TAP-U concordance with humoral response.

Through comparative profiling of peptides presented in OPSCC tissue and healthy tonsils (HT), we found that many of the top class I and II TAP source proteins, such as CTNNB1, TP53, KDMB5, EGFR, and COL6A1, are closely linked to oncogenic pathways previously identified in HNSCC or other cancer entities (19, 20, 58–60). This suggests that proteins promoting and sustaining the oncogenic phenotype might be the richest source of HLA ligands among OPSCC TAP. However, a weak concordance (25%) was found between upregulated mRNA and HLA class I TAP genes, similar to findings by Weinzierl et al. in renal cell carcinoma (61). The discrepancy between TAP density and transcription levels could be due to high protein turnover rates (62), intricate post-transcriptional and translational modifications (51, 52), or the boosted production of defective ribosomal products (DRiPs) (51, 63) from mutated genes, which in turn comprised the majority of top presented TAP in this study.

The low presentation of MAGEA3 peptides and the absence of NY-ESO1 peptides in the ligandome, despite their known protein expression and immunogenicity in HNSCC, suggest that MAGEA3-targeted vaccines may have reduced efficacy (38–42), and highlights a potential challenge in utilizing NY-ESO1 as a target for immunotherapy in OPSCC. Interestingly, several upregulated HLA class I TAP genes (*IGF2BP1*, *FJX1*, *PRAME*, *CCNA1*, and *MAGEA4*) exhibited a significant positive correlation between presentation and expression. Notably, *IGF2BP1* and *FJX1* correlated in HPV+ patients, while *PRAME* and *CCNA1* in HPV- patients. Our previous work linked IGF2BP1 antibodies with poor prognosis in HPV+ patients (49). The immunogenicity of FJX1, when targeted by DNA-based vaccines, leading to efficient tumor clearance independently or with anti-PD1, highlights its potential as a vaccine target (64). Similarly, PRAME previously elicited a robust immune response in leukemia and melanoma (65–69), and CCNA1 was adopted as a vaccine target for acute myeloid leukemia (70).

*MAGEA4*, with the highest gene expression upregulation in our OPSCC cohort, correlated with HLA class I display exclusively in low-stage patients. Vaccination against MAGEA4 in advanced cancers showed safety and potential immunogenicity based on its expression and HLA class I presence (71). A phase I trial investigated the safety, kinetics, and clinical effect of T lymphocytes transduced with a MAGEA4-specific T-cell receptor gene in patients with unresectable and treatment-refractory solid tumors, including HNSCC (72). Although results for this trial have not yet been released, it underscores the ongoing exploration of CTA as promising immunotherapeutic targets.

Furthermore, no correlation was found between mRNA levels and HLA class II presentation of gene-derived ligands. This contrasts with Schuster et al. findings in ovarian carcinomas, which were based on data from a single patient. Despite its limited scope, the latter study remains the sole investigation that assessed the degree of association between ligand presentation and gene expression in cancer context (73). *PRSS50* was the only HLA class II ligand to show a significant correlation between presentation and expression, primarily in HPV+ and low-stage patients. Despite its potential importance, *PRSS50* remains underexplored in cancer research. Notably, it has been identified as differentially expressed in resident and metastatic tumor-educated platelets in patients with colorectal cancer (CRC) and non-small cell lung carcinoma (74).

The identification of tumor-unique peptides (TAP-U) presented on HLA class I and II opens promising avenues for targeted immunotherapies. These peptides can inform novel therapeutic modalities like therapeutic cancer vaccines, ACT, and engineered T cell therapies, enhancing treatment efficacy and minimizing adverse effects. Filtering TAP against 30 benign tissues identified 73 and 62 TAP-U on HLA class I and II, respectively. Notably, eight upregulated genes (*BUB1*, *BUB1B*, *CDK2*, *CDK4*, *FJX1*, *CCNA1*, *MAGEA4*, and *PRSS50*) demonstrated concordance between expression and HLA presentation and high affinity to common HLA haplotypes, making them attractive vaccine candidates or ACT targets for OPSCC.

Top presented HLA class I TAP-U source genes *KIF2C*, *MAGED4*, and *EGFR* have shown immunogenicity in cancer context (64, 75, 76). EGFR TAP-U, linked to poor prognosis in the present study, suggests evaluating its vaccine combination with Cetuximab to improve outcomes. Similarly, LY6K TAP-U presentation on HLA class II was associated with worse prognosis in low-stage patients, potentially due to interactions between APC and CD4+ T cells, which might induce anergy due to the peptide’s length (77, 78). A nonrandomized phase II clinical trial conducted in advanced-stage HNSCC patients resistant to standard therapy evaluated vaccination with peptides derived from CTA LY6K, CDCA1, and IMP3. Vaccinated patients who responded to LY6K or CDCA1 showed significantly longer overall survival compared to non-responders (13).

Examining HLA class II TAP-U revealed no significant association between presentation level and antibody response (AR), suggesting that factors like sampling bias and the transient role of CD4+ T cells in B cell expansion influence quantitative correlations. Interestingly, a recent study revealed that intratumoral B cells in HNSCC display high percentages of activated and antigen-presenting B cells. Nonetheless, tumor-associated antigen responses were linked to distinct patterns of T cell infiltration, with higher antibody levels observed in patients with greater densities of CD4+ T cells (43). This suggests that the lack of concordance may be due to the low infiltration of CD4+ T cells in the tumor. HLA class II TAP-U peptides, with low AR correlation, may induce anti-tumor immune responses when used as vaccines, since antibodies have the dual capacity to opsonize peptides, forming immune complexes, but also to neutralize these peptides, which could attenuate T cell responses during immunizations. Interestingly, BUB1 TAP-U presentation was negatively correlated with AR against most antigens, indicating possible enrichment of presentation on B-regs, possibly impairing effective humoral response (79). FES was the only TAP-U gene showing significant concordance between presentation and AR. Expression of FES was previously shown to be associated with increased tumor proliferation, angiogenesis, the presence of circulating tumor cells and macrophage infiltration, suggesting its diagnostic and recurrence monitoring potential (80).

Pathway analysis of proteins with peptide levels correlating with antigen-presenting cells (APC) infiltration highlighted pathways like focal adhesion and actin cytoskeleton regulation, which may enhance APC efficacy in peptide sampling and presentation (81, 82). Also, enrichment in pathways such as Rap1 and PI3K-Akt signaling, known for regulating immune cell adhesion and motility, further supports their role in optimizing peptide capture by APC (83, 84). The enrichment in pathogen defense pathways suggests these mechanisms might be co-opted by tumors for immune modulation (85, 86). Moreover, the HPV infection pathway’s involvement underscores its role in modulating immune responses (87), with differences being previously observed in CTA expression and related antibody responses in HPV+ versus HPV- patients (23, 41–43).

While this study enhances our understanding of HLA-presented ligands using semiquantitative mass spectrometry technology, further improvements in sensitivity are needed. The novel OPSCC TAP-U identified, though not yet tested for immunogenicity, hold promising potential for future *in vitro* and *in vivo* studies to confirm their therapeutic viability. Additionally, validation in an independent and different population cohort is essential, especially since this study was conducted exclusively using samples from German patients. Future studies should include detailed HLA class II typing to enable more precise predictions of peptide-HLA class II interactions, thereby enhancing the translational impact of our findings. Moreover, it is crucial to investigate the complex mechanisms regulating the flow of information from RNA expression to peptide presentation and the generation of immune responses in OPSCC tumors. These insights will be pivotal for advancing our understanding and application of immunotherapeutic strategies.

## 5 Conclusion

In conclusion, this study demonstrates that relying solely on gene transcript levels would overlook most tumor-associated peptides (TAP) presented on oropharyngeal squamous cell carcinoma (OPSCC) cells. Importantly, the correlation between many upregulated genes and ligand presentation in OPSCC, particularly cancer-testis antigens (CTA), is shown to be dependent on HPV status or tumor stage. While CTA are presented at relatively low levels, novel OPSCC-specific TAP (TAP-U), including many derived from CTA, identified in this study show potential as targets for immunotherapy. These TAP-U can be utilized to develop personalized cancer vaccines that train the immune system to recognize and target tumor cells. Through HLA class I presentation, these vaccines primarily stimulate cytotoxic T cells, and when presented on HLA class II molecules, they can engage helper T cells, thereby enhancing cellular immune responses. Additionally, TAP-U can be employed in adoptive cell transfer therapies to further boost the anti-tumor immune response by expanding and reinfusing T cells specifically trained to target these peptides.

## Data availability statement

The HLA ligandome mass spectrometry raw data is publicly available at the ProteomeXchange Consortium via the PRIDE partner repository (88). The dataset can be accessed using the identifier PXD033383. The gene expression data of the tumors belongs to the publicly available dataset (89) at the Sequence Read Archive (SRA) under the accession number PRJNA967751. The gene expression data of the 6 healthy mucosa samples is deposited to SRA (BioProject ID: PRJNA1069986). WES data is available at the European Genome-Phenome Archive (EGAS00001006477). Antibody expression data generated from the KREX™ functional proteomics CTA array is deposited in the **Supplementary Material** (**Supplementary Table 7**).

## Ethics statement

The studies involving humans were approved by Ulm University: Approval number 222/13; 90/15. The studies were conducted in accordance with the local legislation and institutional requirements. The participants provided their written informed consent to participate in this study.

## Author contributions

TAK: Writing – review & editing, Writing – original draft, Visualization, Software, Methodology, Investigation, Formal analysis, Data curation, Conceptualization. MM: Writing – review & editing, Software, Methodology, Formal analysis, Data curation. LM: Conceptualization, Writing – review & editing, Visualization, Software, Methodology, Investigation, Data curation. AB: Conceptualization, Writing – review & editing, Resources. FO: Conceptualization, Writing – review & editing, Resources. MB: Writing – review & editing, Methodology, Investigation. JT: Writing – review & editing, Software, Methodology. JK: Writing – review & editing, Resources. JD: Writing – review & editing. AvW: Writing – review & editing. LH: Writing – review & editing. JE: Writing – review & editing, Investigation. DH: Writing – review & editing. JBe: Writing – review & editing, Methodology. TB: Writing – review & editing. JG: Writing – review & editing. PS: Writing – review & editing. CB: Writing – review & editing, Methodology. JBl: Writing – review & editing, Methodology. TH: Writing – review & editing, Supervision, Project administration, Funding acquisition. CO: Writing – review & editing, Resources. HK: Writing – review & editing, Resources. HR: Writing – review & editing, Supervision, Resources, Project administration, Methodology, Funding acquisition. JW: Supervision, Resources, Project administration, Methodology, Investigation, Funding acquisition, Conceptualization, Writing – review & editing. SL: Writing – review & editing, Supervision, Resources, Project administration, Methodology, Investigation, Funding acquisition, Conceptualization.
