# Supplementary figures and images for: Multi-omics analysis of overexpressed tumor-associated proteins: gene expression, immunopeptide presentation, and antibody response in oropharyngeal squamous cell carcinoma, with a focus on cancer-testis antigens
Source: Front Immunol. 2024 Jul 29;15:1408173. doi: 10.3389/fimmu.2024.1408173 (PMC11317303; doi:10.3389/fimmu.2024.1408173)

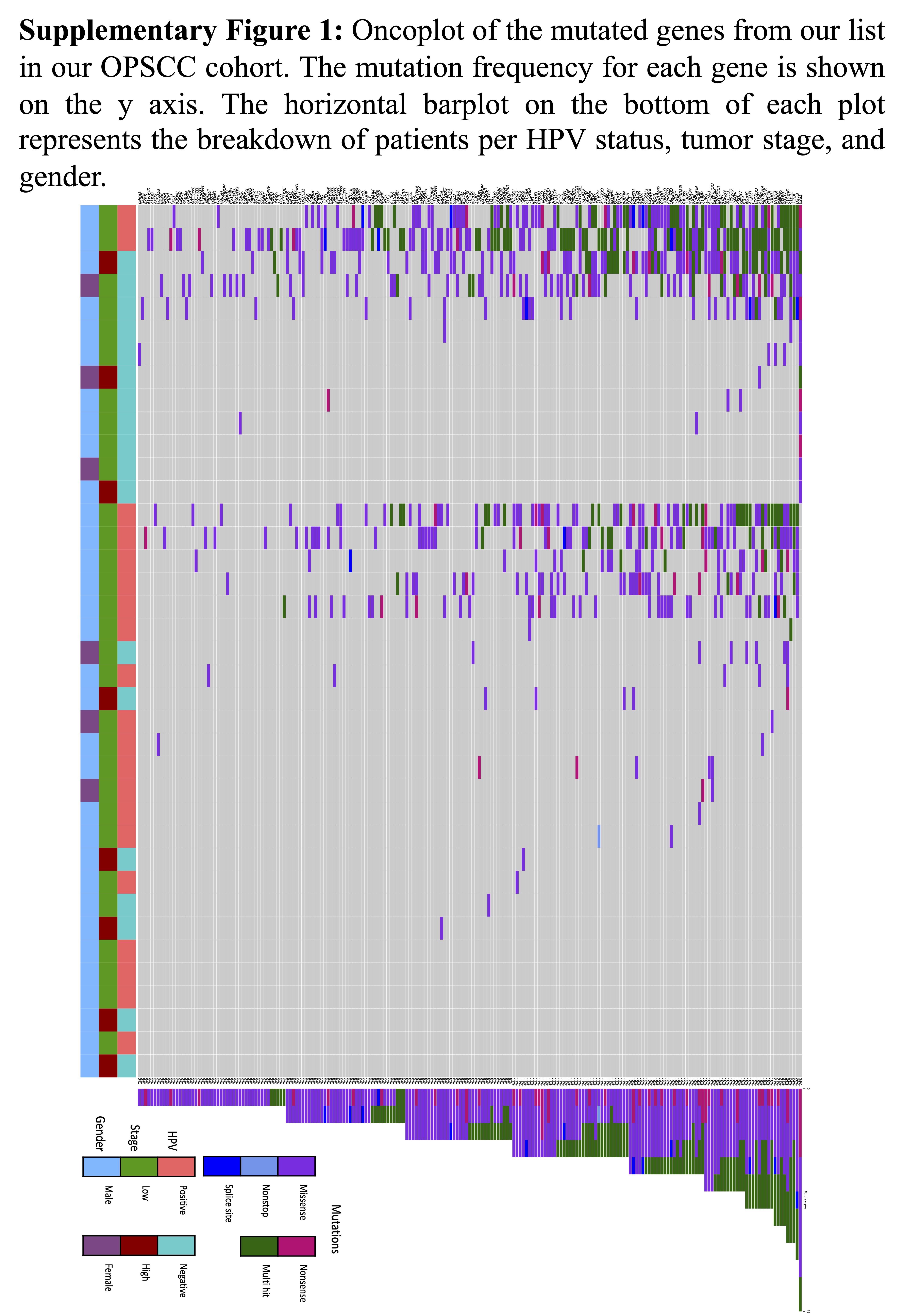

Supplement: Supplementary file 8 [file Image_1.jpeg]

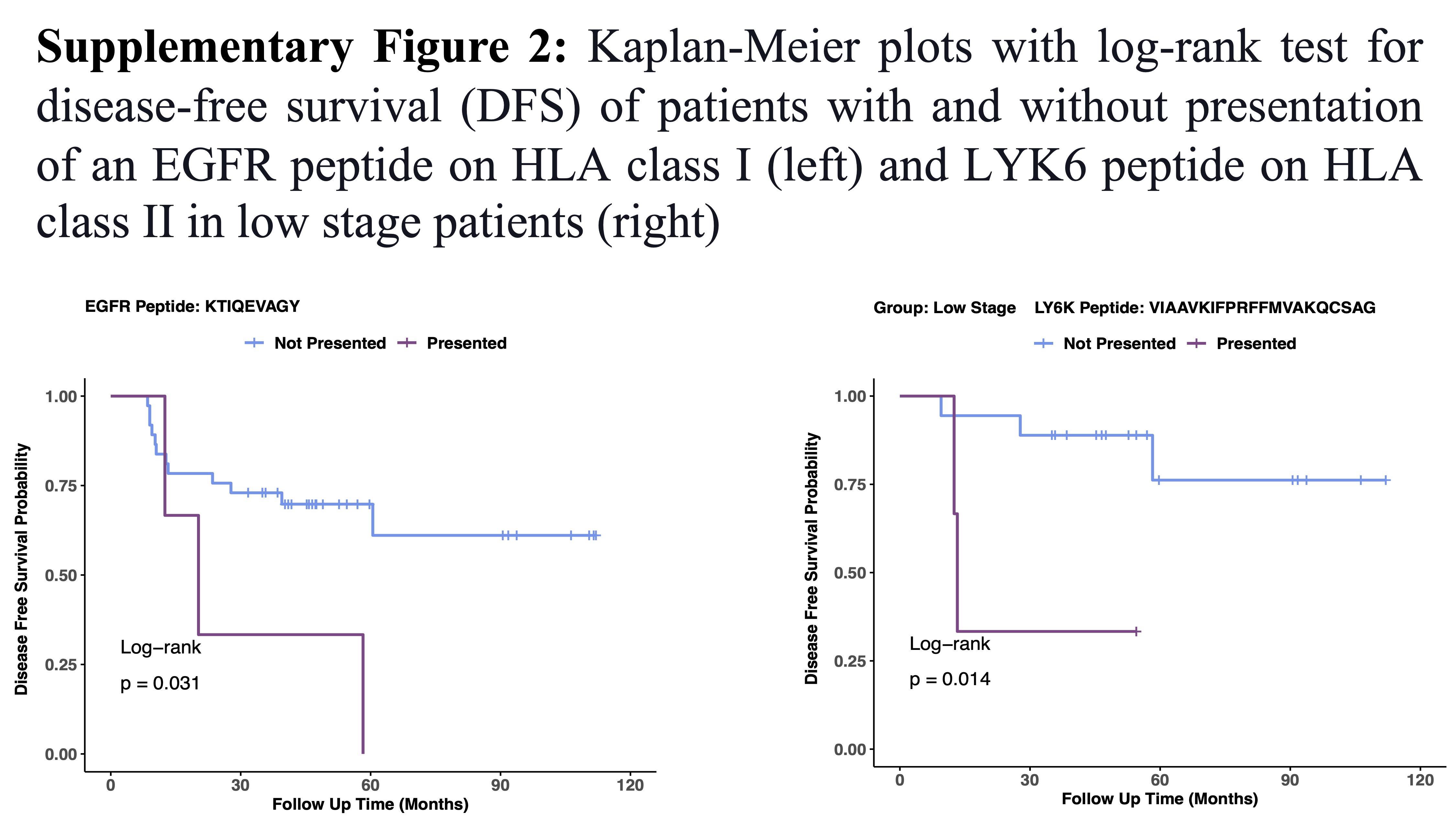

Supplement: Supplementary file 9 [file Image_2.jpeg]

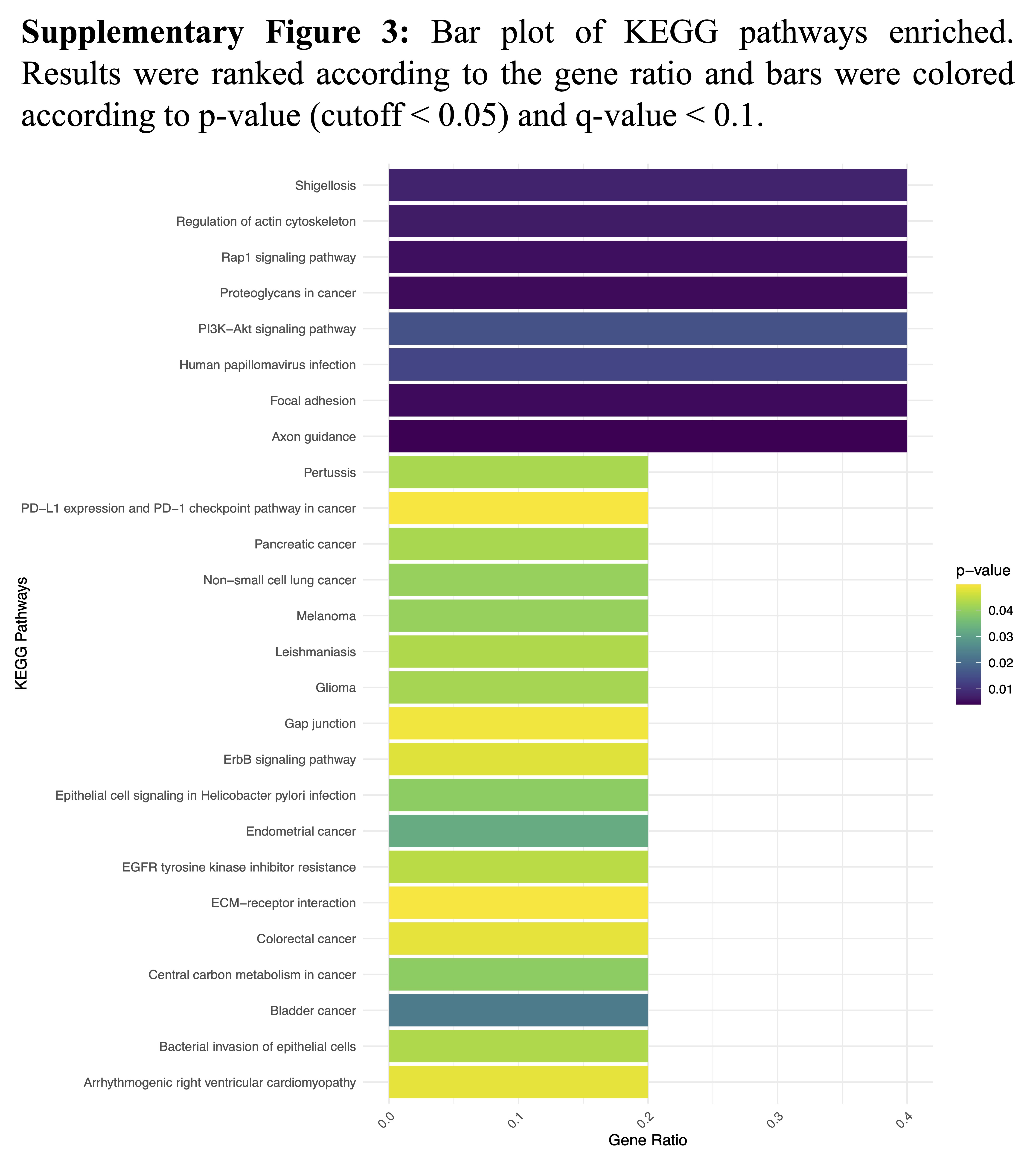

Supplement: Supplementary file 10 [file Image_3.jpeg]
